# Neonatal Hyperoxia Downregulates Claudin-4, Occludin, and ZO-1 Expression in Rat Kidney Accompanied by Impaired Proximal Tubular Development

**DOI:** 10.1155/2020/2641461

**Published:** 2020-12-02

**Authors:** Xuewen Xu, Xinyue Zhang, Linlin Gao, Chunfeng Liu, Kai You

**Affiliations:** ^1^Department of Urology, Shengjing Hospital of China Medical University, Shenyang 110004, China; ^2^Medical Research Center, Shengjing Hospital of China Medical University, Shenyang 110004, China; ^3^Department of Pediatrics, Shengjing Hospital of China Medical University, Shenyang 110004, China

## Abstract

Hyperoxia is essential to manage in preterm infants but causes injury to immature kidney. Previous study indicates that hyperoxia causes oxidative damage to neonatal kidney and impairs renal development. However, the underlying mechanisms by which neonatal hyperoxia effects on immature kidney still need to be elucidated. Tight junction, among which the representative proteins are claudin-4, occludin, and ZO-1, plays a crucial role in nephrogenesis and maintaining renal function. Inflammatory cytokines are involved in the pleiotropic regulation of tight junction proteins. Here, we investigated how neonatal hyperoxia affected the expression of key tight junction proteins and inflammatory factors (IL-6 and TNF-*α*) in the developing rat kidneys and elucidated their correlation with renal injury. We found claudin-4, occludin, and zonula occludens-1 (ZO-1) expression in proximal tubules was significantly downregulated after neonatal hyperoxia. The expression of these tight junction proteins was positively correlated with that of IL-6 and TNF-*α*, while claudin-4 expression was positively correlated with injury score of proximal tubules in mature kidneys. These findings indicated that impaired expression of tight junction proteins in kidney might be a potential mechanism of hyperoxia-induced nephrogenic disorders. It provides new insights to further study oxidative renal injury and development disorders and will be helpful for seeking potential therapeutics for hyperoxia-induced renal injury in the future.

## 1. Introduction

Supplemental oxygen therapy (hyperoxia) is commonly administered in the management of premature infants with respiratory disorders [1, 2]. Nonetheless, increasing evidence from various clinical and experimental observations suggests that neonatal hyperoxia may cause oxidative damage and adversely affects glomerular and tubular maturity [3, 4]. These adverse effects are manifested by enlarged renal corpuscles, renal tubular injuries, and interstitial inflammation during the perinatal period, which might extend into adulthood and influence renal function [5, 6]. However, the exact effects and mechanisms of hyperoxia exposure on immature kidney injury remain unknown.

It is well recognized that intact structures and barriers of tight junctions are required for renal function [7]. Tight junctions, as components of glomerular filtration membranes, play an important role in renal filtration and maintaining glomerular permeability [8]. Tight junctions together with nephrin, known as slit diaphragms, connect adjacent podocyte foot processes in glomeruli [9]. Moreover, tight junction barriers in proximal tubules enable the selective reabsorption of electrolytes and organic nutrients by separating the tubular lumen from the basolateral cell surface [10]. The paracellular permeability of collecting ducts, which play a crucial role in electrolyte reabsorption and secretion, is also mainly regulated by tight junctions [11].

Beyond these important functions in mature kidneys, tight junctions also play an important role in nephrogenesis. In humans, nephrogenesis is lasted until gestation by 32 to 36 weeks, during which preterm neonates were commonly exposed to hyperoxia, whereas in rats, the nephrogenesis proceeds until 5 to 8 days postnatal, which became the sensitive window for the study of rat kidney in response to the hyperoxia exposure [5, 12]. Kidneys undergo development for several months after birth until the adult morphology and size are reached [13]. During this period, the tight junction barrier prevents the basolateral membrane of renal epithelial cells and other structures beneath them from coming into direct contact with filtrates, thus maintaining a microenvironment suitable for nephrogenesis [14].

Accumulating evidence indicates that neonatal hyperoxia can impair nephrogenesis [15, 16]. However, whether neonatal hyperoxia alters tight junction proteins expression in developing kidneys and further affects nephrogenesis remains unclear. Hence, we hypothesized that neonatal hyperoxia could alter tight junction proteins expression in immature kidney and further affect renal development.

Claudin-4, occluding, and ZO-1, as the key components of the tight junction, play critical roles in the normal kidney function [17–19]. Claudin-4 belongs to the claudin family and is encoded by the CLDN4 gene, which is located on chromosome 7. It is an integral membrane protein that is a component of the epithelial cell tight junctions, which regulate the movement of solutes and ions through the paracellular space [17]. Occludin is encoded by the OCLN gene, which is located on chromosome 5 and is required for cytokine-induced regulation of the tight junction paracellular permeability barrier [20]. Occludin is a critical component involved in the hyperpermeability of the glomerular endothelium [18]. ZO-1 is encoded by the TJP1 gene, which is located on chromosome 15 and acts as a tight junction adaptor protein that also regulates adherent junctions. The multidomain structure of this scaffold protein includes a postsynaptic density 95/disc-large/zona occludens (PDZ) domain, a Src homology (SH3) domain, a guanylate kinase (GuK) domain and unique (U) motifs, all coordinate binding of transmembrane proteins, cytosolic proteins, and F-actin, which are required for tight junction function [21].

Inflammatory cytokines are involved in the pleiotropic regulation of tight junction proteins. Interleukin 6 (IL-6) and tumor necrosis factor *α* (TNF-*α*) induce barrier dysfunction of tight junctions [22, 23]. On the other hand, IL-6 and TNF-*α* also have a protective role on the tight junction barrier [24–27]. The regulatory effect of IL-6 and TNF-*α* in the developing kidneys following hyperoxia exposure is still unclear. The purposes of our study were to clarify the effects of neonatal hyperoxia exposure on the expression of key tight junction proteins (claudin-4, occludin, and ZO-1) and inflammatory factors (IL-6 and TNF-*α*) in the developing kidneys and to elucidate their correlation with renal injury and nephrogenesis.

## 2. Materials and Methods

### 2.1. Animal Model

Pregnant Sprague-Dawley rats were purchased from the experimental animal center of Shengjing Hospital of China Medical University. All animal procedures were performed under the approval of the Ethics Committee of Shengjing Hospital, China Medical University (project identification code: 2016PS362K). The animal care and experimental procedures of this study were conducted in compliance with the U.S. Animal Welfare Act and performed in accordance with the standards of the Institute of Laboratory Animal Resources (ILAR) Guide (1996). Pups were born at the gestational age of 21 to 23 days. Within 24 h of birth, all pups were randomly divided into the normoxia group (fraction of inspired air [FiO_2_] = 0.21, *n* = 80) or hyperoxia group ([FiO_2_] = 0.85, *n* = 80) (Supplementary Figure 1). The mothers were rotated between normoxia and hyperoxia exposed litters every 24 h to prevent oxygen toxicity in the dams. The body weight of the animals was recorded every day. Animals were euthanized by an approved method of euthanasia as follows: animals were euthanized by CO2 gas exposure for approximately 5 minutes by first being exposed to 3 L/min of CO2 until the rat was unconscious; then, the flow was escalated to the highest setting to produce more rapid asphyxia. Immediately after euthanasia, the kidney tissues were harvested on the 1st, 3rd, 5th, 7th, 10th, 14th, 30th (exposed to normoxia or hyperoxia in the first 14 days), and 60th (exposed to normoxia or hyperoxia in the first 14 days) postnatal days (*n* = 10/time point/exposure). The left kidneys were fixed in 4% paraformaldehyde (PFA) for hematoxylin and eosin (H&E) staining and immunohistochemical staining, and the right kidneys were kept at -80°C for western blotting.

### 2.2. Kidney Histology

After fixation in 4%PFA for 24 h, kidney samples were dehydrated in gradient ethanol, then embedded in paraffin and longitudinally sectioned (4 *μ*m) for H&E staining. Ten fields of view were randomly chosen and observed at an original magnification of ×400. Histological evaluations were conducted blindly by two pathologists. The evaluation of glomerular diameter was modified according to the suggestions of Toledo-Rodriguez et al. [28]. Digital images of H&E staining were analyzed in the software ImageJ 1.51. The number of grid points occupied by glomeruli was counted. Glomerular diameter was calculated according to the number of grid points occupied by the scale bar. The sizes of the individual glomeruli located in the middle cortex and juxtamedullary zone were calculated as the average of the largest and smallest glomerular diameters within a field of view; the calculations involved ten fields of view per kidney. The significance of tubular injury was determined by evaluation of tubular dilatation, tubular atrophy, vacuolization, degeneration and sloughing of tubular epithelial cells, or thickening of the tubular basement membrane. As previously described, the scoring system used only proximal tubules for tubular injury evaluation, where 0 = no tubular injury, 1 = <10% of tubules injured, 2 = 10–25% of tubules injured, 3 = 26–50% of tubules injured, 4 = 51–75% of tubules injured, and 5 = >75% of tubules injured [29]. The cast counting was performed by quantifying the number of pink-stained structures in the dilated lumen of outer medullary collecting ducts per mm^2^ [30]. The counting involved ten fields of view per sample.

### 2.3. Immunohistochemical Staining

The evaluation of protein expression via immunohistochemical staining was modified according to a previous study [4]. Rat kidney slides were dewaxed, rehydrated, placed in sodium citrate, and microwaved for antigen retrieval. A nonspecific stain blocking agent was added to the slides and incubated for 30 min at room temperature. Primary antibody to claudin-4 (cat. no. 32-9400, 1 : 100, Thermo Fisher Scientific, Waltham, MA), occludin (cat. no. 33-1500, 1 : 200, Thermo Fisher Scientific, Waltham, MA), ZO-1 (cat. no. 33-9100, 1 : 100, Thermo Fisher Scientific, Waltham, MA), CD3 (cat. no. ab11089, 1 : 200, Abcam, Cambridge, USA), CD68 (cat. no. ab201340, 1 : 200, Abcam, Cambridge, USA), nephrin (at. no. ab216341, 1 : 200, Abcam, Cambridge, USA), or Ki-67 (cat. no. ab16667, 1 : 200, Abcam, Cambridge, USA) was added, and slides were incubated overnight at 4°C. After being washed in PBS, the slides were incubated with secondary antibody (Gene Tech, Shanghai, China; dilution 1 : 100) for 30 min at room temperature. Excess secondary antibody was washed off. Slides were incubated with streptavidin–avidin–biotin complex, developed with 3, 3′-diaminobenzidine, washed with running water, counterstained with hematoxylin, dehydrated with gradient alcohol, dried, and mounted with neutral balsam. The results were interpreted blindly by two pathologists. Ten high-power fields of view (original magnification ×400) were randomly selected from each slide, and images were obtained with a light microscope, with 200 cells observed in each field of view. Cells with the cytoplasm or nucleus stained yellow or dark brown were defined as positive cells. Semiquantitative results were obtained by determining the intensity of cell staining in the image-analysis software ImageJ 1.51. The quantification of glomerular number was performed from immunohistochemical staining of nephrin using the ImageJ 1.51 software (Rangan, G.K., Tesch, G.H. 2007. Quantification of renal pathology by image analysis. Nephrology (Carlton) 12 (6):553-558. 10.1111/j.1440-1797.2007.00855.x).

### 2.4. Immunofluorescence Staining

The evaluation of protein expression via immunofluorescence staining was modified according to a previous study [31]. Rat kidney slides were dewaxed, rehydrated, placed in sodium citrate, and microwaved for antigen retrieval. The slides were then blocked with 10% goat serum for 30 min at 37°C and incubated with primary antibodies as follows: N-cadherin (cat. no. PA5-19486, 1 : 200, Thermo Fisher Scientific, Waltham, MA) or ZONAB (ZO-1-associated nucleic acid-binding protein) primary antibody (cat. no. 40-2800, 1 : 100, Thermo Fisher Scientific, Waltham, MA) and ZO-1 primary antibody (cat. no. 33-9100, 1 : 100, Thermo Fisher Scientific, Waltham, MA) together overnight at 4°C. As negative controls, some slides were incubated in the absence of the primary antibodies. Tissue slides were washed four times with PBS-Triton. The second antibody against mouse was conjugated with Alexa fluor-488 (green fluorescence) and the anti-rabbit antibody with Alexa fluor-594 (red fluorescence) for 60 min at room temperature. Slides were washed three times with PBS, and the nuclei were stained with DAPI (1 : 2000; Sigma Chemical) for 2 min. After sufficient washes, images were captured by confocal laser scanning microscopy (MTC-600, BIO-RAD, USA).

### 2.5. Western Blotting

The quantification of protein expression via western blotting was modified according to a previous study [4]. Briefly, an appropriate volume of RIPA buffer was added to kidney tissue samples to produce cell lysates. After centrifugation at 12,000 rpm for 20 min, the protein concentration of each sample supernatant was determined with a bicinchoninic acid protein concentration assay kit (Beyotime, Shanghai, China). A 5× Loading Buffer was used to dilute the protein samples, which were boiled in a water bath for 5 min. Proteins were separated by SDS–polyacrylamide gel electrophoresis and subsequently transferred to a polyvinylidene difluoride membrane (Millipore, Burlington, MA, USA). After incubation with claudin-4 (cat. no. 32-9400, 1 : 1000, Thermo Fisher Scientific, Waltham, MA), occludin (cat. no. 33-1500, 1 : 1000, Thermo Fisher Scientific, Waltham, MA), ZO-1 (cat. no. 33-9100, 1 : 1000, Thermo Fisher Scientific, Waltham, MA), IL-6 (cat. no. DF6087, 1 : 1000, Affinity, OH, USA), TNF-*α* (cat. no. AF7014, 1 : 1000, Affinity, OH, USA) or STAT3 (cat. no. AF6294, 1 : 1000, Affinity, OH, USA) primary antibody and secondary antibodies (1 : 5000), electrochemical luminescence solution (Sigma, St. Louis, MO, USA) was added to produce substrate luminescence. All bands were scanned with the Chemi Imager 5500 V2.03 software (AIPha InnCh, Miami, FL, USA), and integrated density values were calculated with a computerized image analysis system (Fluor Chen 2.0) and normalized to the expression of *β*-actin.

### 2.6. Statistical Analysis

The GraphPad Prism 7 software (GraphPad Software, San Diego, CA, USA) was used for statistical analyses and plotting bar graphs. The results of each assay were obtained after three repeated independent experiments and are expressed as mean ± SEM. One-way analysis of variance (ANOVA) and post hoc comparisons (Bonferroni test) were used to determine significant differences among multiple groups. Simple regression was used to correlate the relative expression detected by western blotting as an independent variable, with the tubular injury score or relative expression detected by western blotting as a dependent variable. A *P* value less than 0.05 was considered statistically significant.

## 3. Results

### 3.1. Neonatal Hyperoxia Downregulates Expression of Claudin-4 in Proximal Tubules

**Figure 1:**
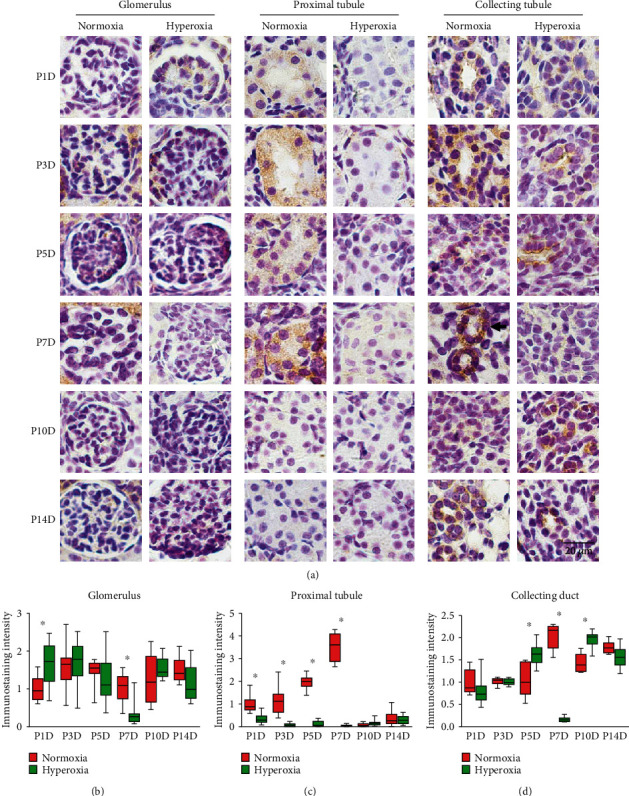
Neonatal hyperoxia downregulates expression of claudin-4 in proximal tubules. (a) Claudin-4 expression in glomeruli, proximal tubules, and collecting ducts of newborn rats, which were exposed to normoxia or hyperoxia from birth to 1st postnatal day (P1D), 3rd postnatal day (P3D), 5th postnatal day (P5D), 7th postnatal day (P7D), 10th postnatal day (P10D), and 14th postnatal day (P14D), was measured, respectively, by immunohistochemical staining (original magnification ×400. Scale bar, 20 *μ*m. Arrow for positive staining). (b–d) The box and whisker plots represent the immunostaining intensity of claudin-4 expression in glomeruli, proximal tubules, and collecting ducts from newborn rats exposed to normoxia or hyperoxia, respectively. Relative expression is standardized to the value of normoxia group on P1D. The whiskers represent the minimal or the maximal intensity, and the boxes span the interquartile range of measurements for 10 rats with the mean value of 3 replicates (*n* = 10). ^∗^*P* < 0.05, one-way ANOVA, Bonferroni post hoc test.

To investigate the expression of claudin-4 in glomeruli, proximal tubules, and collecting ducts after hyperoxia, we performed immunohistochemical staining of kidney tissues from neonatal rats. Mild membranous staining for claudin-4 was observed in glomeruli, and strong cytoplasmic staining was observed in proximal tubules and collecting ducts (Figure 1(a)). Claudin-4 expression in neonatal glomeruli exhibited a double-peak pattern with a trough between the peaks (Figure 1(b)). Significantly lower claudin-4 expression was observed in the hyperoxia group compared with the normoxia group on the 7th postnatal day (normoxia group 1.02 ± 0.40 vs. hyperoxia group 0.37 ± 0.31, *P* < 0.01). However, claudin-4 expression in neonatal glomeruli exposed to hyperoxia was upregulated on the 1st postnatal day (normoxia group 1.0 ± 0.32 vs. hyperoxia group 1.66 ± 0.60, *P* < 0.05; Figure 1(b)). Claudin-4 expression in neonatal proximal tubules reached a peak on the 7th postnatal day and clearly declined after the 10th postnatal day (Figure 1(c)). Claudin-4 expression in proximal tubules was significantly downregulated after hyperoxia on the 1st (normoxia group 1.0 ± 0.38 vs. hyperoxia group 0.34 ± 0.21, *P* < 0.001), 3rd (normoxia group 1.13 ± 0.61 vs. hyperoxia group 0.08 ± 0.08, *P* < 0.001), 5th (normoxia group 1.96 ± 0.30 vs. hyperoxia group 0.12 ± 0.13, *P* < 0.001), and 7th postnatal days (normoxia group 3.54 ± 0.62 vs. hyperoxia group 0.05 ± 0.04, *P* < 0.001), thus indicating that the expression peak of claudin-4 was blunted by hyperoxia in proximal tubules (Figure 1(c)). Claudin-4 expression in neonatal collecting ducts exhibited a double peak with a trough between the peaks, whereas neonatal hyperoxia advanced the time points of these peaks and troughs (Figure 1(d)). Significantly lower claudin-4 expression was found in the hyperoxia group than the normoxia group on the 7th postnatal day (normoxia group 2.06 ± 0.27 vs. hyperoxia group 0.16 ± 0.06, *P* < 0.001; Figure 1(d)). However, claudin-4 expression in neonatal collecting ducts exposed to hyperoxia was upregulated on the 5th and the 10th postnatal days (Figure 1(d)). Therefore, our findings indicate that the expression of claudin-4 was downregulated in proximal tubules by neonatal hyperoxia.

### 3.2. Occludin Expression in Proximal Tubules Is Downregulated by Neonatal Hyperoxia

**Figure 2:**
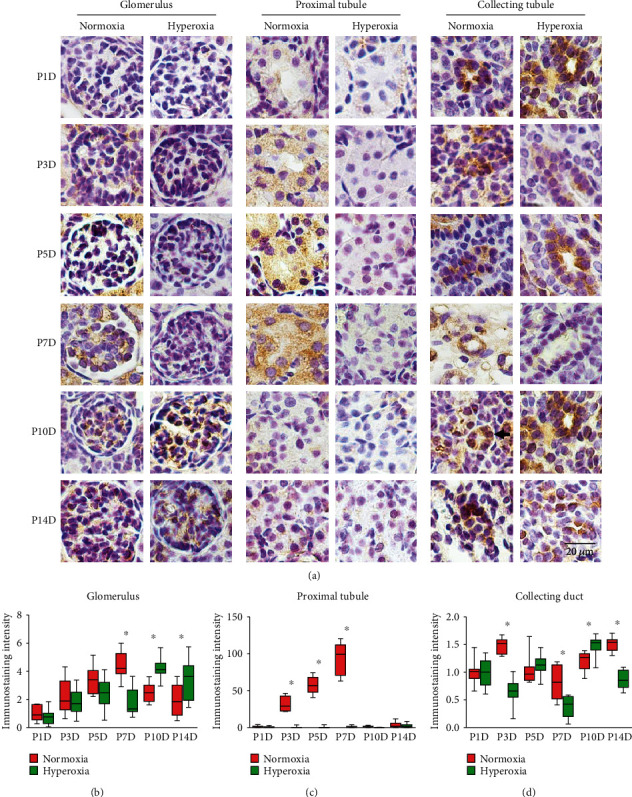
Neonatal hyperoxia downregulates expression of occludin in proximal tubules. (a) Occludin expression in glomeruli, proximal tubules, and collecting ducts of newborn rats, which were exposed to normoxia or hyperoxia from birth to 1st postnatal day (P1D), 3rd postnatal day (P3D), 5th postnatal day (P5D), 7th postnatal day (P7D), 10th postnatal day (P10D), and 14th postnatal day (P14D), was measured, respectively, by immunohistochemical staining (original magnification ×400. Scale bar, 20 *μ*m. Arrow for positive staining). (b–d) The box and whisker plots represent the immunostaining intensity of occludin expression in glomeruli, proximal tubules, and collecting ducts from newborn rats exposed to normoxia or hyperoxia, respectively. Relative expression is standardized to the value of normoxia group on P1D. The whiskers represent the minimal or the maximal intensity, and the boxes span the interquartile range of measurements for 10 rats with the mean value of 3 replicates (*n* = 10). ^∗^*P* < 0.05, one-way ANOVA, Bonferroni post hoc test.

To investigate occludin expression in response to neonatal hyperoxia, we performed immunohistochemical staining of occludin in kidney tissues from neonatal rats. Moderate to strong membranous staining for occludin was observed in glomeruli, and strong cytoplasmic staining was observed in proximal tubules and collecting ducts (Figure 2(a)). A single peak of occludin expression was observed in neonatal glomeruli on the 7th postnatal day, while the expression peak of occludin was postponed until the 10th postnatal day by neonatal hyperoxia (Figure 2(b)). Occludin expression in glomeruli was significantly downregulated after hyperoxia on the 7th postnatal day (normoxia group 4.36 ± 0.91 vs. hyperoxia group 1.73 ± 0.98, *P* < 0.001) and was significantly upregulated after hyperoxia on the 10th (normoxia group 2.49 ± 0.65 vs. hyperoxia group 4.20 ± 0.77, *P* < 0.001) and 14th postnatal days (normoxia group 1.95 ± 1.18 vs. hyperoxia group 3.34 ± 1.42, *P* < 0.01; Figure 2(b)). The expression peak of occludin in neonatal proximal tubules appeared on the 7th postnatal day and was clearly attenuated after the 10th postnatal day (Figure 2(c)). Occludin expression in proximal tubules was significantly downregulated after hyperoxia on the 3rd (normoxia group 31.42 ± 9.96 vs. hyperoxia group 0.43 ± 1.11, *P* < 0.001), 5th (normoxia group 57.54 ± 12.0 vs. hyperoxia group 0.47 ± 1.19, *P* < 0.001), and 7th postnatal days (normoxia group 94.73 ± 20.83 vs. hyperoxia group 1.04 ± 1.33, *P* < 0.001), showing a 91-fold lower expression of occludin in proximal tubules exposed to hyperoxia (Figure 2(c)). Occludin expression in neonatal collecting ducts exhibited a double-peak pattern with a trough between the peaks, whereas neonatal hyperoxia postponed the expression peaks of occludin by 2 or 3 days (Figure 2(d)). Occludin expression in collecting ducts was significantly downregulated after hyperoxia on the 3rd (normoxia group 1.48 ± 0.14 vs. hyperoxia group 0.65 ± 0.24, *P* < 0.001), 7th (normoxia group 0.81 ± 0.29 vs. hyperoxia group 0.38 ± 0.19, *P* < 0.001), and 14th postnatal days (normoxia group 1.50 ± 0.12 vs. hyperoxia group 0.87 ± 0.16, *P* < 0.001) and was significantly upregulated after hyperoxia on the 10th postnatal day (normoxia group 1.20 ± 0.18 vs. hyperoxia group 1.48 ± 0.17, *P* < 0.05; Figure 2(d)). Therefore, the expression of occludin was notably downregulated by neonatal hyperoxia in proximal tubules, and the expression peaks of occludin in glomeruli and collecting ducts were postponed by neonatal hyperoxia.

### 3.3. Neonatal Hyperoxia Blunts the Expression Peak of ZO-1 in Proximal Tubules

**Figure 3:**
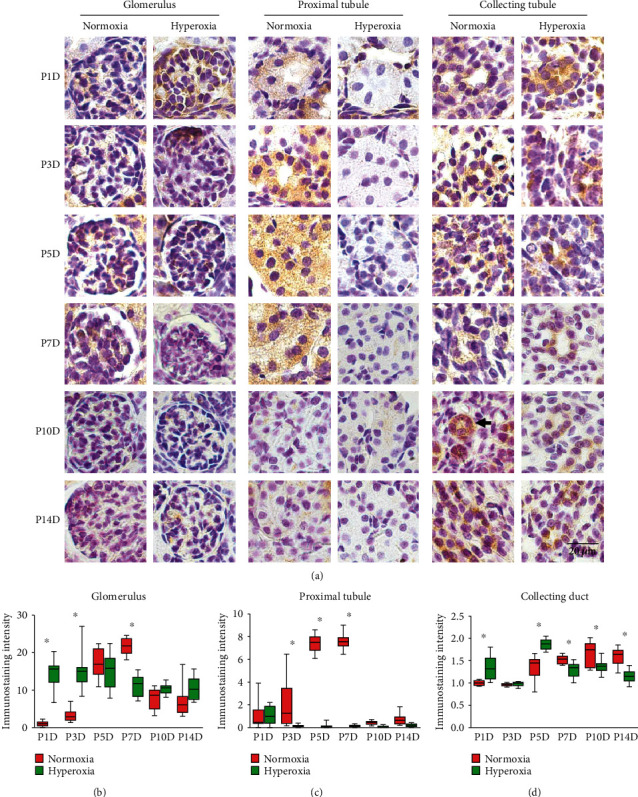
Neonatal hyperoxia downregulates expression of ZO-1 in proximal tubules. (a) ZO-1 expression in glomeruli, proximal tubules, and collecting ducts of newborn rats, which were exposed to normoxia or hyperoxia from birth to 1st postnatal day (P1D), 3rd postnatal day (P3D), 5th postnatal day (P5D), 7th postnatal day (P7D), 10th postnatal day (P10D), and 14th postnatal day (P14D), was measured, respectively, by immunohistochemical staining (original magnification ×400. Scale bar, 20 *μ*m. Arrow for positive staining). (b–d) The box and whisker plots represent the immunostaining intensity of ZO-1 expression in glomeruli, proximal tubules, and collecting ducts from newborn rats exposed to normoxia or hyperoxia, respectively. Relative expression is standardized to the value of normoxia group on P1D. The whiskers represent the minimal or the maximal intensity, and the boxes span the interquartile range of measurements for 10 rats with the mean value of 3 replicates (*n* = 10). ^∗^*P* < 0.05, one-way ANOVA, Bonferroni post hoc test.

The expression of ZO-1 in glomeruli, proximal tubules, and collecting ducts of neonatal rats was measured by immunohistochemical staining. Moderate membranous staining for ZO-1 in glomeruli, and strong cytoplasmic staining for ZO-1, was observed in proximal tubules and collecting ducts (Figure 3(a)). A single peak of ZO-1 expression on the 7th postnatal day was observed in neonatal glomeruli, whereas neonatal hyperoxia advanced the peak of ZO-1 expression, which appeared on the 5th postnatal day (Figure 3(b)). ZO-1 expression in glomeruli was significantly upregulated after hyperoxia on the 1st (normoxia group 1.0 ± 0.69 vs. hyperoxia group 14.50 ± 3.80, *P* < 0.001) and 3rd postnatal days (normoxia group 3.19 ± 1.72 vs. hyperoxia group 15.07 ± 4.93, *P* < 0.001) and was significantly downregulated after hyperoxia on the 7th postnatal day (normoxia group 21.7 ± 2.26 vs. hyperoxia group 11.35 ± 2.88, *P* < 0.001; Figure 3(b)). ZO-1 expression in neonatal proximal tubules peaked on the 7th postnatal day and markedly declined after the 10th postnatal day (Figure 3(c)), which is consistent with the expression patterns of claudin-4 and occludin in proximal tubules. ZO-1 expression in proximal tubules was significantly downregulated after hyperoxia on the 3rd (normoxia group 1.99 ± 1.98 vs. hyperoxia group 0.15 ± 0.10, *P* < 0.001), 5th (normoxia group 7.42 ± 0.76 vs. hyperoxia group 0.12 ± 0.20, *P* < 0.001), and 7th postnatal days (normoxia group 7.57 ± 0.69 vs. hyperoxia group 0.15 ± 0.11, *P* < 0.001), thus indicating that peak expression of ZO-1 was blunted by hyperoxia in proximal tubules (Figure 3(c)). A single peak of ZO-1 expression on the 10th postnatal day was observed in neonatal collecting ducts, and the peak expression of ZO-1 was advanced by neonatal hyperoxia to the 5th postnatal day (Figure 3(d)). ZO-1 expression in collecting ducts was significantly downregulated after hyperoxia on the 7th (normoxia group 1.53 ± 0.09 vs. hyperoxia group 1.30 ± 0.16, *P* < 0.05), 10th (normoxia group 1.64 ± 0.29 vs. hyperoxia group 1.37 ± 0.15, *P* < 0.01), and 14th postnatal days (normoxia group 1.59 ± 0.19 vs. hyperoxia group 1.14 ± 0.14, *P* < 0.001) and was significantly upregulated after hyperoxia on the 1st (normoxia group 1.0 ± 0.06 vs. hyperoxia group 1.35 ± 0.27, *P* < 0.001) and 5th postnatal days (normoxia group 1.35 ± 0.29 vs. hyperoxia group 1.87 ± 0.12, *P* < 0.001; Figure 3(d)). Thus, the expression peak of ZO-1 in proximal tubules was blunted by neonatal hyperoxia, and the expression peaks of ZO-1 in glomeruli and collecting ducts were advanced by neonatal hyperoxia. A high similarity among the changes in expression of claudin-4, occludin, and ZO-1 was observed in the proximal tubules of neonatal rats exposed to hyperoxia. A weak coexpression of ZO-1 and N-cadherin (proximal tubular marker) was observed by immunofluorescence costaining in the glomerular cell membrane on P5D of both normoxia and hyperoxia groups, and cytoplasmic coexpression of ZO-1 and N-cadherin in the proximal tubules was observed on P5D of both normoxia and hyperoxia groups (Supplementary Figure 2(a)). Localization to the cell membrane of ZO-1 expression in proximal tubules was attenuated by hyperoxia exposure on P14D (Supplementary Figure 2(a)). A weak coexpression of ZO-1 and ZONAB (ZO-1-associated nucleic acid-binding protein) was observed in the glomerular cell membrane on P5D of both normoxia and hyperoxia groups, and cytoplasmic coexpression of ZO-1 and ZONAB in the proximal tubules was observed on P5D of both normoxia and hyperoxia groups (Supplementary Figure 2(b)). A strong expression of ZONAB was observed in the glomerular cell membrane on P14D of both normoxia and hyperoxia groups (Supplementary Figure 2(b)). Nephrin (a podocyte marker) expression in glomeruli was significantly downregulated after hyperoxia on the 14th postnatal day (normoxia group 1.70 ± 0.50 vs. hyperoxia group 0.93 ± 0.07, *P* < 0.001). The cytoplasmic expression of Ki-67 (a cell proliferation marker) in proximal tubules was significantly downregulated after hyperoxia on the 14th postnatal day (normoxia group 2.08 ± 0.32 vs. hyperoxia group 1.04 ± 0.38, *P* < 0.001).

### 3.4. The Expression of Tight Junction Proteins Is Positively Correlated with That of Inflammatory Cytokines in Rat Kidneys

**Figure 4:**
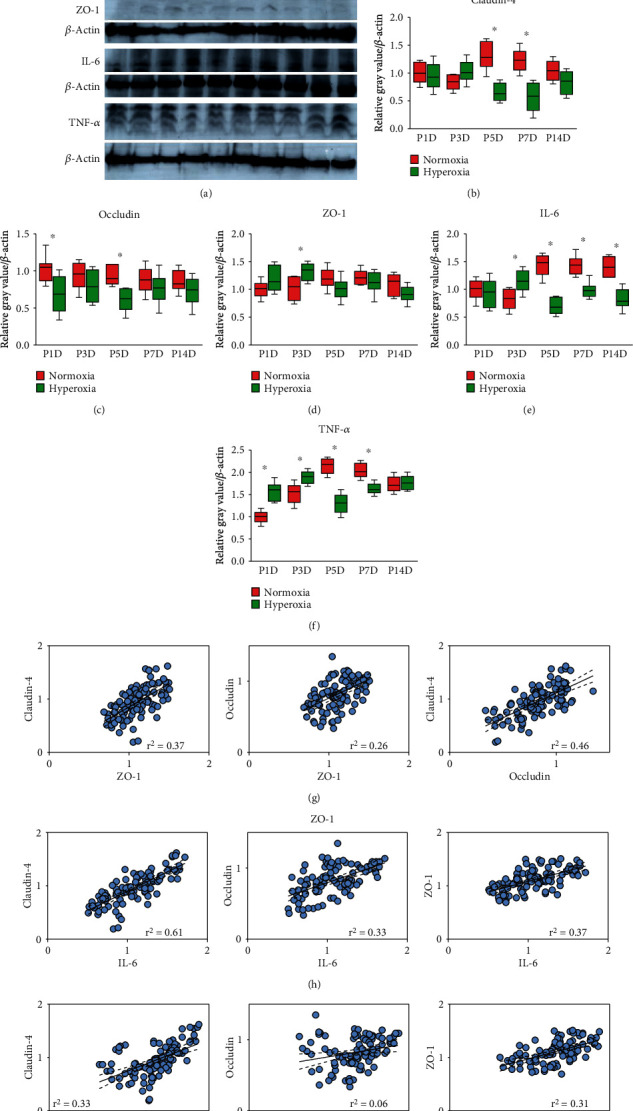
Tight junction protein expression is positively correlated with that of inflammatory cytokines after neonatal hyperoxia. (a) Expression bands of claudin-4, occludin, ZO-1, IL-6, and tumor necrosis factor-*α* (TNF-*α*) in the kidneys of newborn rats exposed to normoxia or hyperoxia till 1st postnatal day (P1D), 3rd postnatal day (P3D), 5th postnatal day (P5D), 7th postnatal day (P7D), and 14th postnatal day (P14D) were detected by western blotting. (b–f) The box and whisker plots represent relative protein expression of claudin-4, occludin, ZO-1, IL-6, and TNF-*α*, respectively. Quantified band intensities were normalized to *β*-actin and then standardized to the value of normoxia group on P1D. The whiskers represent the minimal or the maximal gray value, and the boxes span the interquartile range of measurements for 10 rats with the mean value of 3 replicates (*n* = 10). ^∗^*P* < 0.05, one-way ANOVA, Bonferroni post hoc test. (g) Graphs represent linear correlation among expression of claudin-4, occludin, and ZO-1. (h) Graphs represent linear correlation between expression of claudin-4, occludin, ZO-1, and IL-6. (i) Graphs represent linear correlation between expression of claudin-4, occludin, ZO-1, and TNF-*α* in neonatal kidneys exposed to hyperoxia or normoxia. The correlation coefficient (*r*^2^) along with the best-fit line (solid line) and 95% confidence band (dashed line) is plotted when it is found to be statistically significant (*n* = 20, simple regression).

To evaluate inflammatory cytokine production after hyperoxia and to quantify the total expression of tight junction proteins in rat kidneys exposed to neonatal hyperoxia, we examined the expression of claudin-4, occludin, ZO-1, IL-6, and TNF-*α* in the kidneys of newborn rats by western blotting (Figure 4(a)). Claudin-4 expression peaked on the 5th postnatal day, and this effect was blunted by hyperoxia (Figures 4(a) and 4(b)). Total claudin-4 expression was significantly downregulated after hyperoxia on the 5th (normoxia group 1.32 ± 0.24 vs. hyperoxia group 0.66 ± 0.15, *P* < 0.001) and 7th postnatal days (normoxia group 1.23 ± 0.19 vs. hyperoxia group 0.57 ± 0.26, *P* < 0.001; Figures 4(a) and 4(b)). Total occludin expression was significantly downregulated after hyperoxia on the 1st (normoxia group 1.02 ± 0.16 vs. hyperoxia group 0.69 ± 0.24, *P* < 0.001) and 5th postnatal days (normoxia group 0.94 ± 0.13 vs. hyperoxia group 0.61 ± 0.1, *P* < 0.001; Figures 4(a) and 4(c)). Total ZO-1 expression in neonatal kidneys was not significantly altered after hyperoxia except for the 3rd postnatal day (normoxia group 1.02 ± 0.20 vs. hyperoxia group 1.32 ± 0.15, *P* < 0.01; Figures 4(a) and 4(d)). The expression of inflammatory cytokines after hyperoxia presented a biphase pattern, thus indicating that they were upregulated before the 3rd postnatal day and downregulated after the 5th postnatal day. The expression of IL-6 was significantly upregulated after hyperoxia on the 3rd postnatal day (normoxia group 0.83 ± 0.18 vs. hyperoxia group 1.53 ± 0.20, *P* < 0.001) and significantly downregulated after hyperoxia on the 5th (normoxia group 1.43 ± 0.19 vs. hyperoxia group 0.69 ± 0.14, *P* < 0.001), 7th (normoxia group 1.43 ± 0.17 vs. hyperoxia group 0.98 ± 0.13, *P* < 0.001), and 14th postnatal days (normoxia group 1.41 ± 0.17 vs. hyperoxia group 0.82 ± 0.17, *P* < 0.001; Figures 4(a) and 4(e)). TNF-*α* expression was significantly upregulated after hyperoxia on the 1st (normoxia group 1.0 ± 0.13 vs. hyperoxia group 1.57 ± 0.19, *P* < 0.001) and 3rd postnatal days (normoxia group 1.52 ± 0.22 vs. hyperoxia group 1.89 ± 0.13, *P* < 0.001) and was significantly downregulated after hyperoxia on the 5th (normoxia group 2.15 ± 0.16 vs. hyperoxia group 1.31 ± 0.21, *P* < 0.001) and 7th postnatal days (normoxia group 2.03 ± 0.16 vs. hyperoxia group 1.63 ± 0.12, *P* < 0.001; Figures 4(a) and 4(f)). Thus, inflammatory cytokine production increased before the 3rd postnatal day and decreased after the 5th postnatal day.

To further investigate the correlation between the expression of tight junction proteins and that of inflammatory cytokines, we performed simple regression using the relative expression values of tight junction proteins and inflammatory cytokines, which were detected by western blotting. For tight junction proteins, positive correlations were found between ZO-1 and claudin-4 (*r*^2^ = 0.37, *P* < 0.001), occludin and ZO-1 (*r*^2^ = 0.26, *P* < 0.001), and occludin and claudin-4, respectively (*r*^2^ = 0.46, *P* < 0.001; Figure 4(g)). There were positive correlations between IL-6 and claudin-4 (*r*^2^ = 0.61, *P* < 0.001), and occludin (*r*^2^ = 0.33, *P* < 0.001) and ZO-1 expression (*r*^2^ = 0.37, *P* < 0.001; Figure 4(h)). Positive correlations were found between the expression of TNF-*α* and claudin-4 (*r*^2^ = 0.33, *P* < 0.001), and occludin (*r*^2^ = 0.06, *P* < 0.05) and ZO-1 (*r*^2^ = 0.31, *P* < 0.001; Figure 4(i)). Thus, these results indicated positive correlations between the expression of tight junction proteins and inflammatory cytokines. Despite the expression of IL-6 and TNF-*α* in both normoxia or hyperoxia groups, the T cell (CD3 positive) or macrophage (CD68 positive) was absent either in the glomeruli or proximal tubules measured by immunohistochemical staining (Supplementary Figures 4(a) and 4(b)). The STAT3 (signal transducer and activator of transcription 3) expression in the kidneys of neonatal rats, in response to the downregulation of inflammatory factors, was significantly downregulated after hyperoxia on the 1st (normoxia group 1.02 ± 0.06 vs. hyperoxia group 0.82 ± 0.05, *P* < 0.001), 3rd (normoxia group 1.17 ± 0.03 vs. hyperoxia group 0.97 ± 0.06, *P* < 0.001), 5th (normoxia group 1.19 ± 0.05 vs. hyperoxia group 1.03 ± 0.04, *P* < 0.001), and 14th postnatal days (normoxia group 1.10 ± 0.06 vs. hyperoxia group 0.89 ± 0.05, *P* < 0.001; Supplementary Figure 4(c)).

### 3.5. Neonatal Hyperoxia Downregulates Claudin-4 and Occludin in Collecting Ducts as well as ZO-1 in Proximal Tubules of Adult Rats

**Figure 5:**
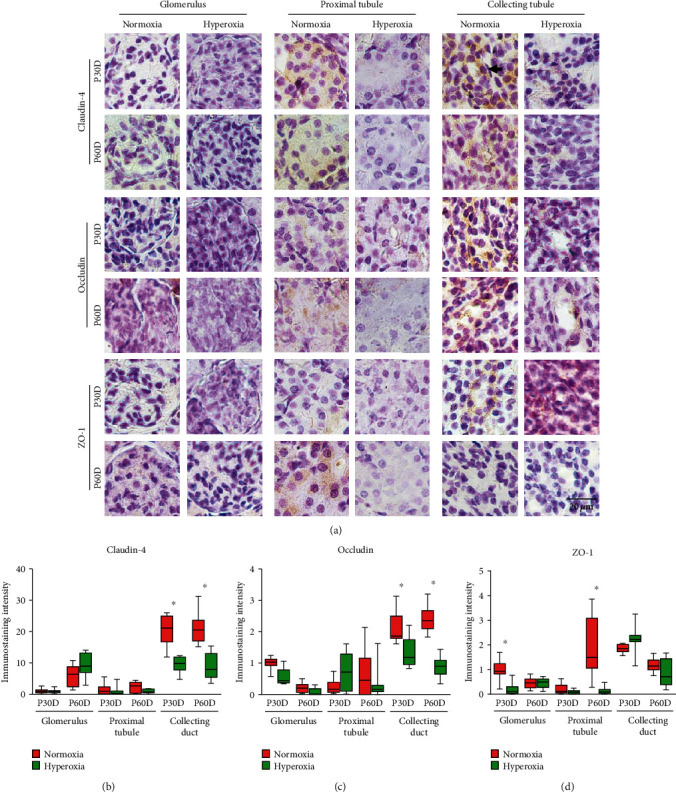
Neonatal hyperoxia downregulates claudin-4 in proximal tubules as well as occludin and ZO-1 in the collecting ducts of adult rats. (a) The expression of claudin-4, occludin, and ZO-1 in glomeruli, proximal tubules, and collecting ducts of adult rats on 30th postnatal day (P30D) and 60th postnatal day (P60D), which were exposed to normoxia or hyperoxia from birth to 14th postnatal day, was measured, respectively, by immunohistochemical staining (original magnification ×400. Scale bar, 20 *μ*m. Arrow for positive staining). (b–d) Graphs represent the immunostaining intensity of claudin-4, occludin, and ZO-1 expression in glomeruli, proximal tubules, and collecting ducts of adult rats on P30D and P60D, which were exposed to normoxia or hyperoxia from birth to 14th postnatal day. Relative expression is standardized to the value of normoxia group on P30D. Values are means ± SE from *n* = 10 samples. ^∗^*P* < 0.05 compared with normoxia group (one-way ANOVA).

To investigate the long-term effects of neonatal hyperoxia on the expression of tight junction proteins in rat kidneys, we determined the expression of claudin-4, occludin, and ZO-1 in the kidneys of adult rats on the 30th and 60th postnatal days by immunohistochemical staining. For claudin-4, mild membranous or cytoplasmic staining in glomeruli and proximal tubules was observed, whereas strong cytoplasmic staining in collecting ducts was observed (Figure 5(a)). For occludin, mild cytoplasmic staining was observed in glomeruli, moderate cytoplasmic staining was observed in proximal tubules, and strong cytoplasmic staining was observed in collecting ducts from adult rats (Figure 5(a)). For ZO-1, mild cytoplasmic staining in glomeruli and moderate cytoplasmic staining in proximal tubules and collecting ducts were found in mature kidneys (Figure 5(a)). Claudin-4 expression in the collecting ducts of adult rats was significantly downregulated by neonatal hyperoxia on the 30th (normoxia group 20.77 ± 4.61 vs. hyperoxia group 9.39 ± 2.48, *P* < 0.001) and 60th postnatal days (normoxia group 20.94 ± 4.95 vs. hyperoxia group 9.12 ± 4.12, *P* < 0.001; Figure 5(b)). Occludin expression in the collecting ducts of adult rats was significantly downregulated by neonatal hyperoxia on the 30th (normoxia group 2.10 ± 0.49 vs. hyperoxia group 1.34 ± 0.46, *P* < 0.001) and 60th postnatal days (normoxia group 2.42 ± 0.47 vs. hyperoxia group 0.89 ± 0.32, *P* < 0.001; Figure 5(c)). ZO-1 expression in glomeruli of adult rats was significantly downregulated by neonatal hyperoxia on the 30th postnatal day (normoxia group 1.0 ± 0.40 vs. hyperoxia group 0.19 ± 0.24, *P* < 0.001), whereas that in the proximal tubules of adult rats was significantly downregulated by neonatal hyperoxia on the 60th postnatal day (normoxia group 1.95 ± 1.20 vs. hyperoxia group 0.14 ± 0.15, *P* < 0.001; Figure 5(d)). Therefore, neonatal hyperoxia downregulated claudin-4 and occludin in collecting ducts as well as ZO-1 in the proximal tubules of adult rats, thus showing that neonatal hyperoxia has a long-term inhibitory role toward tight junction proteins in rat kidneys.

### 3.6. The Injury Score of Proximal Tubules Is Negatively Correlated with Claudin-4 Expression in Mature Kidneys

**Figure 6:**
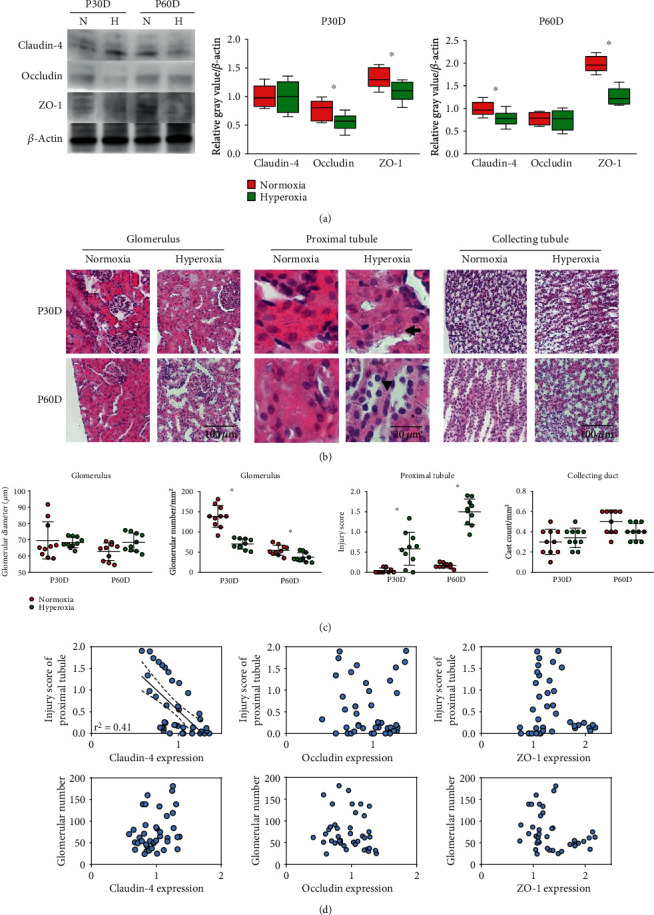
The injury score of proximal tubules is negatively correlated with claudin-4 expression in mature kidneys. (a) Expression bands of claudin-4, occludin, and ZO-1 in kidneys of adult rats on 30th postnatal day (P30D) and 60th postnatal day (P60D), which were exposed to neonatal normoxia or hyperoxia from birth to 14th postnatal day, were detected by western blotting. The whisker and box plots represent relative protein expression of claudin-4, occludin, and ZO-1. Quantified band intensities were normalized to *β*-actin and then standardized to the value of normoxia group on P30D. The whiskers represent the minimal or the maximal gray value, and the boxes span the interquartile range of measurements for 10 rats with the mean value of 3 replicates (*n* = 10). ^∗^*P* < 0.05, one-way ANOVA, Bonferroni post hoc test. (b) The glomeruli, proximal tubules, and collecting ducts of adult rats, including those from the 30th postnatal day (P30D) and 60th postnatal day (P60D), which were exposed to neonatal normoxia or hyperoxia from birth to 14th postnatal day were detected by hematoxylin and eosin (H&E) staining (original magnification ×400. Scale bar, 100 *μ*m or 20 *μ*m. Arrow for vacuolation, and arrowheads for thinner tubules and tubular dilation). (c) Graphs represent the glomerular diameters, glomerular numbers, injury scores of proximal tubules, and cast counts in collecting ducts from adult rats exposed to neonatal normoxia or hyperoxia from birth to 14th postnatal day. Values are means ± SE from *n* = 10 samples. ^∗^*P* < 0.05 compared with normoxia group (one-way ANOVA). (d) Graphs represent linear correlation between relative immunostaining intensity of claudin-4, occludin, ZO-1, injury scores of proximal tubules, and glomerular number in mature kidneys exposed to neonatal hyperoxia or normoxia from birth to 14th postnatal day. The correlation coefficient (*r*^2^) along with the best-fit line (solid line) and 95% confidence band (dashed line) is plotted when it is found to be statistically significant (*n* = 20, simple regression).

To validate the results of immunohistochemical staining in the kidneys of adult rats, we determined the expression of claudin-4, occludin, and ZO-1 in the kidneys of adult rats by western blotting (Figure 6(a)). On the 30th postnatal day, occludin (normoxia group 0.78 ± 0.17 vs. hyperoxia group 0.56 ± 0.14, *P* < 0.05) and ZO-1 expression (normoxia group 1.33 ± 0.18 vs. hyperoxia group 1.09 ± 0.18, *P* < 0.05) was significantly downregulated by neonatal hyperoxia (Figure 6(a)). On the 60th postnatal day, claudin-4 (normoxia group 1.0 ± 0.17 vs. hyperoxia group 0.78 ± 0.15, *P* < 0.05) and ZO-1 (normoxia group 1.98 ± 0.18 vs. hyperoxia group 1.26 ± 0.19, *P* < 0.001) was significantly downregulated by neonatal hyperoxia (Figure 6(a)). Thus, the expression of tight junction proteins in rat kidneys was inhibited by neonatal hyperoxia in the long term, findings consistent with the observations from immunohistochemical staining.

To investigate the effects of hyperoxia-induced impairment of kidney development, we performed a histologic examination with H&E staining, using mature kidney samples (30th and 60th postnatal days) from the normoxia and hyperoxia groups (Figure 6(b)). Increased vacuoles, irregular brush-like epithelial borders, and lumen dilation were observed in the proximal tubules of adult rats in the hyperoxia group (Figure 6(b)). The difference in the glomerular diameter of adult rats between the normoxia and hyperoxia group was not significant (Figure 6(c), Table 1), thus indicating that neonatal hyperoxia did not impair glomerular development. Neonatal hyperoxia significantly decreased the glomerular number of adult rats on the 30th (normoxia group 138.4 ± 27.1/mm^2^ vs. hyperoxia group 70.0 ± 12.8/mm^2^, *P* < 0.001) and 60th postnatal days (normoxia group 54.6 ± 12.5/mm^2^ vs. hyperoxia group 37.3 ± 11.4/mm^2^, *P* < 0.001; Figure 6(c), Table 1). Neonatal hyperoxia significantly increased the injury score of proximal tubules of adult rats on the 30th (normoxia group 0.1 ± 0.1 vs. hyperoxia group 0.6 ± 0.4, *P* < 0.001) and 60th postnatal days (normoxia group 0.2 ± 0.1 vs. hyperoxia group 1.5 ± 0.3, *P* < 0.001; Figure 6(c), Table 1). Because severe injury to tubular epithelial cells can cause increased cast formation in collecting ducts, we determined the cast count in the collecting ducts of adult rats by using H&E staining of sections. The difference in cast count in collecting ducts of adult rats between the normoxia and hyperoxia groups was not significant (Figure 6(c), Table 1). Our results indicated that neonatal hyperoxia impaired proximal tubular development in the long term.

To further investigate the correlation between the expression of tight junction proteins and proximal tubular injury, we performed simple regression using the relative expression values of tight junction proteins detected by western blotting and the injury score of proximal tubules on the 30th and 60th postnatal days in adult rats. A negative correlation was found between claudin-4 expression and the injury score of proximal tubules (*r*^2^ = 0.41, *P* < 0.001; Figure 6(d)), whereas no significant correlation between occludin or ZO-1 expression and the injury score in proximal tubules was observed (*P* > 0.05; Figure 6(d)). No significant correlation between claudin-4, occludin, or ZO-1 expression and the glomerular number was observed (*P* > 0.05; Figure 6(d)). Thus, the injury score of proximal tubules was negatively correlated with claudin-4 expression in mature kidneys of adult rats.

## 4. Discussion

Increasing evidence indicates that neonatal hyperoxia causes injury to immature kidney [4, 16, 32]. Since tight junction plays a crucial role in nephrogenesis and maintaining renal function [33, 34] and hyperoxia-induced developmental impairment is mediated by breakdown of the tight junction [31, 35, 36], identifying how neonatal hyperoxia affects tight junction in developing kidneys is of interest. Herein, to our knowledge, this present study showed for the first time that the expression of key tight junction proteins claudin-4, occludin, and ZO-1 in proximal tubules was significantly downregulated after neonatal hyperoxia. The expression of these tight junction proteins was positively correlated with that of IL-6 and TNF-*α*. Furthermore, our study demonstrated that neonatal hyperoxia decreased glomerular number and the proliferation of proximal tubular cells and that the extent of proximal tubular injury was negatively correlated with the expression of claudin-4 in the kidneys of adult rats.

Claudin-4 is a critical component in regulating paracellular chloride permeability of the collecting duct [37, 38]. Claudin-4 also accumulates claudin-1 and claudin-3 in tight junctions to enhance cell-cell contact in renal tubular epithelial cells [39]. We found that claudin-4 expression in glomeruli was altered by neonatal hyperoxia; however, neonatal hyperoxia did not affect claudin-4 expression in glomeruli of adult rats. Our study provides the first demonstration that neonatal hyperoxia downregulates proximal tubular claudin-4 expression, which is negatively correlated with the injury score of proximal tubules of adult rats, thus indicating that neonatal hyperoxia might impair proximal tubular development through downregulation of claudin-4. Importantly, we observed that neonatal hyperoxia downregulated claudin-4 in the collecting ducts of adult rats. Since claudin-4 is a critical component involved in chloride reabsorption in collecting ducts [38], we postulate that neonatal hyperoxia might impair chloride reabsorption in collecting ducts.

Occludin is an endothelial cell marker in glomerular vasculature and plays an important role in the hyperpermeability of the glomerular endothelium, which is regulated by TNF-*α* [8, 40]. In the current study, neonatal hyperoxia postulated the expression peaks of occludin in glomeruli, and the expression of occludin was positively correlated to TNF-*α*, thus suggesting that neonatal hyperoxia might interfere with the filtration function of developing glomeruli in a TNF-*α*-dependent manner. Occludin is an important component in maintaining paracellular permeability of the proximal tubule and collecting duct [41, 42]. We found that neonatal hyperoxia notably downregulated the expression of occludin in proximal tubules and collecting ducts of adult rats, indicating that neonatal hyperoxia is a long-term risk factor for the paracellular permeability of the proximal tubule and collecting duct.

ZO-1, as the marker of tight junction, is a scaffold protein which cross-links and anchors tight junction strand proteins to the actin cytoskeleton [43]. We observed a cytoplasmic expression of ZO-1 in proximal tubule of neonatal rats, and the membranous localization of ZO-1 expression was attenuated by neonatal hyperoxia, indicating that neonatal hyperoxia might impair the membranous anchoring of tight junction proteins by downregulation of membranous ZO-1 in the proximal tubule. ZO-1 and ZONAB interactions are critical components of a tight junction-associated signal transduction pathway regulating the transition of proximal tubular cells from a proliferative to a differentiated phenotype, and cytoplasmic expression of ZONAB is important for cell survival [44, 45]. Membranous coexpression of ZO-1 and ZONAB in glomerulus and cytoplasmic coexpression of those in the proximal tubule of both normoxia and hyperoxia groups was observed in this study, indicating that ZO-1 and ZONAB might mainly involve in the tubular proliferation and cell survival and glomerular differentiation of neonatal rats. Our current study demonstrated that neonatal hyperoxia downregulated ZO-1 expression in both neonatal and adult proximal tubules, which might impair the proliferation of proximal tubular cells during nephrogenesis. Podocyte-specific deletion of the ZO-1 gene impairs slit diaphragm formation, thus leading to abnormal filtration of developing glomeruli [19]. We observed that the expression peaks of ZO-1 in glomeruli were advanced, and nephrin (a marker of podocyte) expression was downregulated by neonatal hyperoxia, which might interfere with the filtration of developing glomeruli. In the current study, the expression peaks of ZO-1 in collecting ducts were advanced by neonatal hyperoxia. Because suppression of ZO-1 resulted in increased G0/G1 retention in the principal cells of collecting ducts [46], hyperoxia-induced alteration of ZO-1 expression in the collecting ducts of neonatal rats may interfere with the cell division of the developing collecting duct. We observed positive correlations among claudin-4, occludin, and ZO-1 expression in neonatal kidneys, thus suggesting that coordinated expression of these tight junction proteins is required during nephrogenesis.

We observed that IL-6 and TNF-*α* in rat kidneys were downregulated after neonatal hyperoxia, indicating that IL-6 and TNF-*α* might be involved in the regulatory mechanism of impaired nephrogenesis. Both IL-6 and TNF-*α* are pleiotropic cytokines that have been implicated in various forms of renal disease [47, 48]. TNF-*α* regulates IL-6 expression via STAT3 signaling, which promotes both stem cell self-renewal and differentiation into tubular cells [49, 50]. IL-6 also triggers mesenchymal-epithelial transition, which is critical for kidney development [51, 52]. We observed that hyperoxia exposure significantly downregulated STAT3 expression accompanied by downregulation of IL-6 and TNF-*α* in the kidney of neonatal rats, indicating synergistic downregulation of IL-6 and TNF-*α* by hyperoxia exposure might be mediated by STAT3 signaling.

IL-6 and TNF-*α* enhance the cell junction of the kidney through MAPK (mitogen-activated protein kinase) signaling [53, 54]. Our study observed a downregulation of TNF-*α* after neonatal hyperoxia and a positive correlation among TNF-*α* and claudin-4, occludin, and ZO-1 in the rat kidneys, indicating that hyperoxia-induced downregulation of the tight junction proteins might be mediated by downregulation of IL-6 and TNF-*α* during nephrogenesis. The observation above is consistent with the result of a previous study, showing that neonatal hyperoxia downregulated the MAPK signaling, which is critical to the nephrogenesis by regulating cell cycle arrest, progenitor maintenance, and differentiation [4, 55, 56]. We found less podocyte, less glomerular number, attenuated proliferation of proximal tubular cells (Ki-67 downregulation), and increased injury score of proximal tubules accompanied by the downregulation of IL-6 and TNF-*α* following neonatal hyperoxia, indicating an impaired kidney development by neonatal hyperoxia exposure might be mediated by IL-6 and TNF-*α*. Interestingly, CD3 and CD68 expression of the rat kidney in response to downregulation of IL-6 and TNF-*α* following neonatal hyperoxia was evaluated in this study, and the T cell (CD3 positive) or macrophage (CD68 positive) was absent either in the glomeruli or proximal tubules was observed. The observation above demonstrated that IL-6 and TNF-*α* were involved in the impaired nephrogenesis by neonatal hyperoxia independent of their proinflammatory effect.

## 5. Conclusion

Our present study showed for the first time that neonatal hyperoxia extensively inhibited the expression of tight junction proteins throughout the development of neonatal proximal tubules, in which inflammatory cytokines might function coordinately. In the long term, neonatal hyperoxia decreased glomerular number and increased injury score of proximal tubules in mature kidneys, which might be an effect mediated by downregulation of claudin-4. These findings indicated that impaired expression of tight junction proteins in kidney might be a potential mechanism of hyperoxia-induced nephrogenic disorders. It provides new insights to further study oxidative renal injury and development disorders and will be helpful for seeking potential therapeutics for hyperoxia-induced renal injury in the future.

## Figures and Tables

**Table 1:** Comparison of renal morphology^a^ of adult rats between neonatal normoxia and hyperoxia exposure.

Parameter	Normoxia	Hyperoxia	*P* value
Glomerular diameter, *μ*m			
P30D	69.6 ± 11.4	68.3 ± 3.3	>0.05
P60D	62.7 ± 5.5	68.4 ± 5.6	>0.05
Glomerular number, per mm^2^			
P30D	138.4 ± 27.1	70.0 ± 12.8	<0.001
P60D	54.6 ± 12.5	37.3 ± 11.4	<0.001
Injury score of the proximal tubule			
P30D	0.1 ± 0.1	0.6 ± 0.4	<0.001
P60D	0.2 ± 0.1	1.5 ± 0.3	<0.001
Cast count, per mm^2^			
P30D	0.3 ± 0.1	0.3 ± 0.1	>0.05
P60D	0.5 ± 0.1	0.4 ± 0.1	>0.05

Note: ^a^measured by hematoxylin and eosin staining; P30D: postnatal 30th day; P60D: postnatal 60th day.

## Data Availability

The datasets during the current study are available from the corresponding authors upon request.

## Abbreviations

ZO-1: Zonula occludens-1
IL-61: Interleukin 6
TNF-*α*1: Tumor necrosis factor *α*
FiO_2_1: Oxygen fraction of inspired air
PFA1: Paraformaldehyde
MAPK1: Mitogen-activated protein kinase
STAT31: Signal transducer and activator of transcription 3
ZONAB1: ZO-1-associated nucleic acid-binding protein.

## Supplementary Materials

Supplementary Materials Supplementary Figure 1: flowchart of study design and animal groups. Newborn rats were exposed to normoxia or hyperoxia and were euthanized on the 1st postnatal day (P1D), 3rd postnatal day (P3D), 5th postnatal day (P5D), 7th postnatal day (P7D), 10th postnatal day (P10D), 14th postnatal day (P14D), 30th postnatal day (P30D), and 60th postnatal day (P60D). The kidneys were harvested immediately following euthanasia for assays of histological examination, immunohistochemical staining (IHC), immunofluorescence staining, and western blotting. Body weight (BW) was recorded as mean ± SD g. Supplementary Figure 2: hyperoxia exposure attenuated membranous localization of ZO-1 in proximal tubules. (a) The distribution of N-cadherin and ZO-1 expression in glomeruli and proximal tubules of newborn rats, which were exposed to normoxia or hyperoxia from birth to 5th postnatal day (P5D) and 14th postnatal day (P14D), was measured, respectively, by immunofluorescence costaining (original magnification ×800. Scale bar, 40 *μ*m. Arrow for membranous localization of ZO-1). (b) The distribution of ZO-1-associated nucleic acid-binding protein (ZONAB) and ZO-1 expression in glomeruli and proximal tubules of newborn rats, which were exposed to normoxia or hyperoxia from birth to 5th postnatal day (P5D) and 14th postnatal day (P14D), was measured, respectively, by immunofluorescent staining (original magnification ×800. Scale bar, 40 *μ*m. Arrowheads for membranous localization of ZONAB). Supplementary Figure 3: neonatal hyperoxia downregulates expression of nephrin in glomeruli and that of Ki-67 in proximal tubules. Nephrin (a) and Ki-67 (b) expression in glomeruli and proximal tubules of newborn rats, which were exposed to normoxia or hyperoxia from birth to 5th postnatal day (P5D) and 14th postnatal day (P14D), was measured, respectively, by immunohistochemical staining (original magnification ×400. Scale bar, 20 *μ*m). The box and whisker plot represents the immunostaining intensity of expression in glomeruli and proximal tubules. Relative expression is standardized to the value of normoxia group on P5D. The whiskers represent the minimal or the maximal intensity, and the boxes span the interquartile range of measurements for 10 rats with the mean value of 3 replicates (*n* = 10). ^∗^*P* < 0.05, one-way ANOVA, Bonferroni post hoc test. Supplementary Figure 4 There was an absence of CD3 and CD68 positive cells in the glomeruli and proximal tubules of normoxia or hyperoxia groups. CD3 (a) and CD68 (b) expression in glomeruli and proximal tubules of newborn rats, which were exposed to normoxia or hyperoxia from birth to 5th postnatal day (P5D) and 14th postnatal day (P14D), was measured, respectively, by immunohistochemical staining (original magnification ×400. Scale bar, 20 *μ*m). The box and whisker plot represents the immunostaining intensity of expression in glomeruli and proximal tubules. Relative expression is standardized to the value of normoxia group on P5D. The whiskers represent the minimal or the maximal intensity, and the boxes span the interquartile range of measurements for 10 rats with the mean value of 3 replicates (*n* = 10). ^∗^*P* < 0.05, one-way ANOVA, Bonferroni post hoc test. (c) Expression bands of STAT3 in the kidneys of newborn rats exposed to normoxia or hyperoxia from birth to 1st postnatal day (P1D), 3rd postnatal day (P3D), 5th postnatal day (P5D), 7th postnatal day (P7D), and 14th postnatal day (P14D) were detected by western blotting. Relative expression adjusted to the reference gene *β*-actin and then standardized to the value of normoxia group on P1D is shown by box and whisker plots. The whiskers represent the minimal or the maximal gray value, and the boxes span the interquartile range of measurements for 10 rats with the mean value of 3 replicates (*n* = 10). ^∗^*P* < 0.05, one-way ANOVA, Bonferroni post hoc test.

## References

[1] Cherian S., Morris I., Evans J., Kotecha S. (2014). Oxygen therapy in preterm infants. *Paediatric Respiratory Reviews*.

[2] Zoban P. (2019). Optimal oxygen saturation in extremely premature neonates. *Physiological Research*.

[3] Mohr J., Voggel J., Vohlen C. (2019). IL-6/Smad2 signaling mediates acute kidney injury and regeneration in a murine model of neonatal hyperoxia. *The FASEB Journal*.

[4] Xu X., You K., Bu R. (2019). Proximal tubular development is impaired with downregulation of MAPK/ERK signaling, HIF-1*α*, and catalase by hyperoxia exposure in neonatal rats. *Oxidative Medicine and Cellular Longevity*.

[5] Popescu C. R., Sutherland M. R., Cloutier A. (2013). Hyperoxia exposure impairs nephrogenesis in the neonatal rat: role of HIF-1*α*. *PLoS One*.

[6] Sutherland M. R., O'Reilly M., Kenna K. (2013). Neonatal hyperoxia: effects on nephrogenesis and long-term glomerular structure. *American Journal of Physiology. Renal Physiology*.

[7] Balkovetz D. F. (2009). Tight junction claudins and the kidney in sickness and in health. *Biochimica et Biophysica Acta*.

[8] Luo P.-L., Wang Y.-J., Yang Y.-Y., Yang J.-J. (2018). Hypoxia-induced hyperpermeability of rat glomerular endothelial cells involves HIF-2*α* mediated changes in the expression of occludin and ZO-1. *Brazilian Journal of Medical and Biological Research*.

[9] Lennon R., Randles M. J., Humphries M. J. (2014). The importance of podocyte adhesion for a healthy glomerulus. *Frontiers in Endocrinology*.

[10] Vedula E. M., Alonso J. L., Arnaout M. A., Charest J. L. (2017). A microfluidic renal proximal tubule with active reabsorptive function. *PLoS One*.

[11] Hinze C., Ruffert J., Walentin K. (2018). GRHL2 is required for collecting duct epithelial barrier function and renal osmoregulation. *Journal of the American Society of Nephrology*.

[12] Yzydorczyk C., Comte B., Cambonie G. (2008). Neonatal oxygen exposure in rats leads to cardiovascular and renal alterations in adulthood. *Hypertension*.

[13] Saint-Faust M., Boubred F., Simeoni U. (2014). Renal development and neonatal adaptation. *American Journal of Perinatology*.

[14] Denker B. M., Sabath E. (2011). The biology of epithelial cell tight junctions in the kidney. *Journal of the American Society of Nephrolog*.

[15] Nakagawa M., Nishizaki N., Endo A. (2017). Impaired nephrogenesis in neonatal rats with oxygen-induced retinopathy. *Pediatrics International*.

[16] Sutherland M. R., Beland C., Lukaszewski M. A., Cloutier A., Bertagnolli M., Nuyt A. M. (2016). Age- and sex-related changes in rat renal function and pathology following neonatal hyperoxia exposure. *Physiological Reports*.

[17] Gong Y., Hou J. (2017). Claudins in barrier and transport function-the kidney. *Pflügers Archiv - European Journal of Physiology*.

[18] Cheng Z., Qi R., Li L. (2018). Dihydroartemisinin ameliorates sepsis-induced hyperpermeability of glomerular endothelium via up-regulation of occludin expression. *Biomedicine & Pharmacotherapy*.

[19] Itoh M., Nakadate K., Horibata Y. (2014). The structural and functional organization of the podocyte filtration slits is regulated by Tjp1/ZO-1. *PLoS One*.

[20] Cong X., Zhang Y., Yang N. Y. (2013). Occludin is required for TRPV1-modulated paracellular permeability in the submandibular gland. *Journal of Cell Science*.

[21] Nomme J., Fanning A. S., Caffrey M., Lye M. F., Anderson J. M., Lavie A. (2011). The Src homology 3 domain is required for junctional adhesion molecule binding to the third PDZ domain of the scaffolding protein ZO-1. *The Journal of Biological Chemistry*.

[22] Kim K. Y., Oh T. W., Do H. J. (2018). Acer palmatum thumb. Ethanol extract alleviates interleukin-6-induced barrier dysfunction and dextran sodium sulfate-induced colitis by improving intestinal barrier function and reducing inflammation. *Journal of Immunology Research*.

[23] Bercier P., Grenier D. (2019). TNF-*α* disrupts the integrity of the porcine respiratory epithelial barrier. *Research in Veterinary Science*.

[24] Mitazaki S., Honma S., Suto M. (2011). Interleukin-6 plays a protective role in development of cisplatin-induced acute renal failure through upregulation of anti-oxidative stress factors. *Life Sciences*.

[25] Huang L., Zhang R., Wu J. (2011). Increased susceptibility to acute kidney injury due to endoplasmic reticulum stress in mice lacking tumor necrosis factor-*α* and its receptor 1. *Kidney International*.

[26] Gangwar R., Meena A. S., Shukla P. K. (2017). Calcium-mediated oxidative stress: a common mechanism in tight junction disruption by different types of cellular stress. *The Biochemical Journal*.

[27] Lee M. S., Su T. C., Huang Y. C. (2018). Effects of vitamin B-6 supplementation on oxidative stress and inflammatory response in neonatal rats receiving hyperoxia therapy. *Journal of Food and Drug Analysis*.

[28] Toledo-Rodriguez M., Loyse N., Bourdon C., Arab S., Pausova Z. (2012). Effect of prenatal exposure to nicotine on kidney glomerular mass and AT1R expression in genetically diverse strains of rats. *Toxicology Letters*.

[29] Jiang J. S., Chou H. C., Yeh T. F., Chen C. M. (2015). Neonatal hyperoxia exposure induces kidney fibrosis in rats. *Pediatrics and Neonatology*.

[30] De Miguel C., Das S., Lund H., Mattson D. L. (2010). T lymphocytes mediate hypertension and kidney damage in Dahl salt-sensitive rats. *American Journal of Physiology. Regulatory, Integrative and Comparative Physiology*.

[31] You K., Xu X., Fu J. (2012). Hyperoxia disrupts pulmonary epithelial barrier in newborn rats via the deterioration of occludin and ZO-1. *Respiratory Research*.

[32] Gubhaju L., Sutherland M. R., Black M. J. (2011). Preterm birth and the kidney: implications for long-term renal health. *Reproductive Sciences*.

[33] Khairallah H., El Andalousi J., Simard A. (2014). Claudin-7, -16, and -19 during mouse kidney development. *Tissue Barriers*.

[34] Minuth M., Schiller A., Taugner R. (1981). The development of cell junction during nephrogenesis. *Anatomy and Embryology*.

[35] Porzionato A., Guidolin D., Macchi V. (2016). Fractal analysis of alveolarization in hyperoxia-induced rat models of bronchopulmonary dysplasia. *American Journal of Physiology. Lung Cellular and Molecular Physiology*.

[36] Xu S., Xue X., You K., Fu J. (2016). Caveolin-1 regulates the expression of tight junction proteins during hyperoxia-induced pulmonary epithelial barrier breakdown. *Respiratory Research*.

[37] Hou J., Renigunta A., Yang J., Waldegger S. (2010). Claudin-4 forms paracellular chloride channel in the kidney and requires claudin-8 for tight junction localization. *Proceedings of the National Academy of Sciences of the United States of America*.

[38] Gong Y., Yu M., Yang J. (2014). The Cap1-claudin-4 regulatory pathway is important for renal chloride reabsorption and blood pressure regulation. *Proceedings of the National Academy of Sciences of the United States of America*.

[39] Ikari A., Atomi K., Takiguchi A. (2012). Enhancement of cell-cell contact by claudin-4 in renal epithelial Madin-Darby canine kidney cells. *Journal of Cellular Biochemistry*.

[40] Sun Z., Xie Q., Pan J., Niu N. (2019). Cadmium regulates von Willebrand factor and occludin expression in glomerular endothelial cells of mice in a TNF-*α*-dependent manner. *Renal Failure*.

[41] Kim S., Kim G. H. (2017). Roles of claudin-2, ZO-1 and occludin in leaky HK-2 cells. *PLoS One*.

[42] Lee S. Y., Shin J. A., Kwon H. M., Weiner I. D., Han K. H. (2011). Renal ischemia-reperfusion injury causes intercalated cell-specific disruption of occludin in the collecting duct. *Histochemistry and Cell Biology*.

[43] Itoh M., Nagafuchi A., Moroi S., Tsukita S. (1997). Involvement of ZO-1 in cadherin-based cell adhesion through its direct binding to alpha catenin and actin filaments. *The Journal of Cell Biology*.

[44] Pozzi A., Zent R. (2010). ZO-1 and ZONAB interact to regulate proximal tubular cell differentiation. *Journal of the American Society of Nephrology*.

[45] Nie M., Balda M. S., Matter K. (2012). Stress- and Rho-activated ZO-1-associated nucleic acid binding protein binding to p 21 mRNA mediates stabilization, translation, and cell survival. *Proceedings of the National Academy of Sciences of the United States of America*.

[46] Qiao X., Roth I., Feraille E., Hasler U. (2014). Different effects of ZO-1, ZO-2 and ZO-3 silencing on kidney collecting duct principal cell proliferation and adhesion. *Cell Cycle*.

[47] Liu Q., Imaizumi T., Aizawa T. (2019). Cytosolic sensors of viral RNA are involved in the production of interleukin-6 via toll-like receptor 3 signaling in human glomerular endothelial cells. *Kidney & Blood Pressure Research*.

[48] Qing X., Chinenov Y., Redecha P. (2018). iRhom2 promotes lupus nephritis through TNF-*α* and EGFR signaling. *The Journal of Clinical Investigation*.

[49] Tanabe K., Matsushima-Nishiwaki R., Yamaguchi S., Iida H., Dohi S., Kozawa O. (2010). Mechanisms of tumor necrosis factor-*α*-induced interleukin-6 synthesis in glioma cells. *Journal of Neuroinflammation*.

[50] Chen T. C., Neupane M., Chien S. J. (2019). Characterization of adult canine kidney epithelial stem cells that give rise to dome-forming tubular cells. *Stem Cells and Development*.

[51] Barasch J., Yang J., Ware C. B. (1999). Mesenchymal to epithelial conversion in rat metanephros is induced by LIF. *Cell*.

[52] Zhang L., Wu T., Qiao S. (2019). miR-1 and miR-802 regulate mesenchymal-epithelial transition during kidney development by regulating Wnt-4/*β*-catenin signaling. *American Journal of Translational Research*.

[53] Patrick D. M., Leone A. K., Shellenberger J. J., Dudowicz K. A., King J. M. (2006). Proinflammatory cytokines tumor necrosis factor-alpha and interferon-gamma modulate epithelial barrier function in Madin-Darby canine kidney cells through mitogen activated protein kinase signaling. *BMC Physiology*.

[54] HO A., WONG C., LAM C. (2008). Tumor necrosis factor-alpha up-regulates the expression of CCL2 and adhesion molecules of human proximal tubular epithelial cells through MAPK signaling pathways. *Immunobiology*.

[55] Smeeton J., Zhang X., Bulus N. (2010). Integrin-linked kinase regulates p38 MAPK-dependent cell cycle arrest in ureteric bud development. *Development*.

[56] Ihermann-Hella A., Hirashima T., Kupari J. (2018). Dynamic MAPK/ERK activity sustains nephron progenitors through niche regulation and primes precursors for differentiation. *Stem Cell Reports*.

