# Correction to “Neonatal Hyperoxia Downregulates Claudin‐4, Occludin, and ZO‐1 Expression in Rat Kidney Accompanied by Impaired Proximal Tubular Development”

**DOI:** 10.1155/omcl/9871853

**Published:** 2026-02-12

X. Xu, X. Zhang, L. Gao, C. Liu, and K. You, “Neonatal Hyperoxia Downregulates Claudin‐4, Occludin, and ZO‐1 Expression in Rat Kidney Accompanied by Impaired Proximal Tubular Development,” *Oxidative Medicine and Cellular Longevity* 2020, no. 1 (2020): 1–18, https://doi.org/10.1155/2020/2641461.

In the article titled “Neonatal Hyperoxia Downregulates Claudin‐4, Occludin, and ZO‐1 Expression in Rat Kidney Accompanied by Impaired Proximal Tubular Development,” there was an error in Figure 2a, which resulted in the wrong image appearing for “occludin expression in collecting duct of P1D hyperoxia,” which was a duplication of the image for “occludin expression in collecting duct of P10D hyperoxia.” This error occurred during figure assembly and should be corrected as follows:

Figure 2Neonatal hyperoxia downregulates expression of occludin in proximal tubules. (a) Occludin expression in glomeruli, proximal tubules, and collecting ducts of newborn rats, which were exposed to normoxia or hyperoxia from birth to 1st postnatal day (P1D), 3rd postnatal day (P3D), 5th postnatal day (P5D), 7th postnatal day (P7D), 10th postnatal day (P10D), and 14th postnatal day (P14D), was measured, respectively, by immunohistochemical staining (original magnification ×400. Scale bar, 20 μm. Arrow for positive staining). (b–d) The box and whisker plots represent the immunostaining intensity of occludin expression in glomeruli, proximal tubules, and collecting ducts from newborn rats exposed to normoxia or hyperoxia, respectively. Relative expression is standardized to the value of normoxia group on P1D. The whiskers represent the minimal or the maximal intensity, and the boxes span the interquartile range of measurements for 10 rats with the mean value of 3 replicates (*n* = 10).  ^∗^ *P* < 0.05, one‐way ANOVA, Bonferroni post hoc test.

**Figure:**
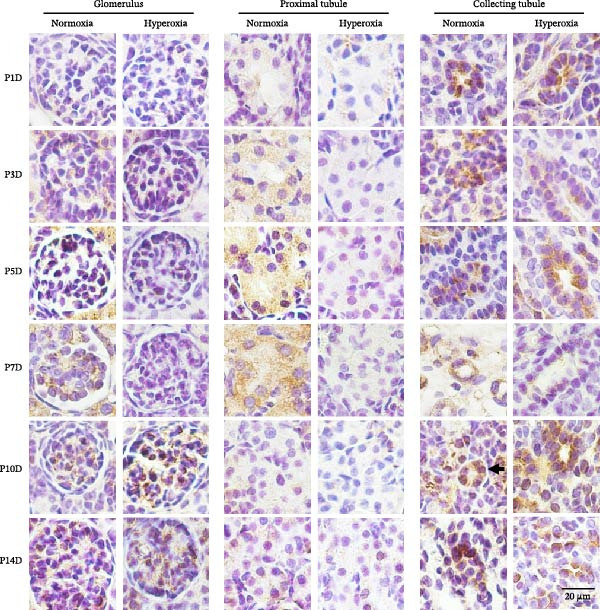
(a)

**Figure:**
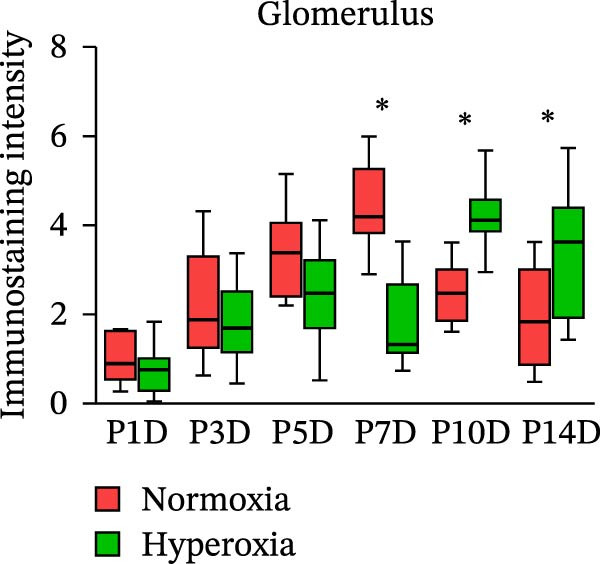
(b)

**Figure:**
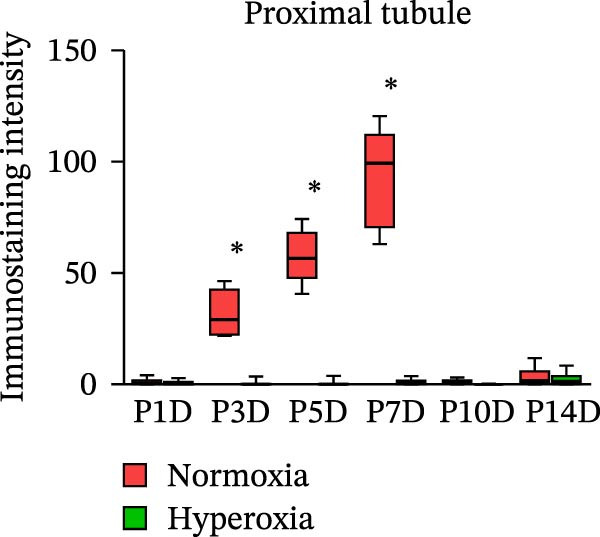
(c)

**Figure:**
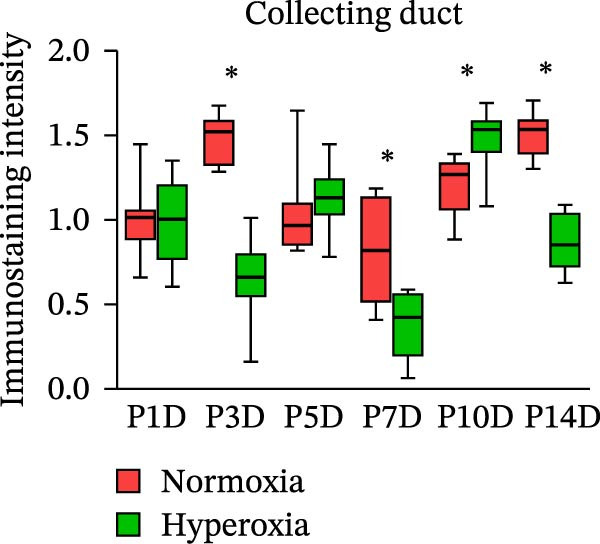
(d)

We apologize for this error.

