# Supplementary material for: Neonatal Hyperoxia Downregulates Claudin-4, Occludin, and ZO-1 Expression in Rat Kidney Accompanied by Impaired Proximal Tubular Development
Source: Oxid Med Cell Longev. 2020 Dec 2;2020:2641461. doi: 10.1155/2020/2641461 (PMC7725566; doi:10.1155/2020/2641461)
Supplement: Supplementary Materials — Supplementary Figure 1: flowchart of study design and animal groups. Newborn rats were exposed to normoxia or hyperoxia and were euthanized on the 1st postnatal day (P1D), 3rd postnatal day (P3D), 5th postnatal day (P5D), 7th postnatal day (P7D), 10th postnatal day (P10D), 14th postnatal day (P14D), 30th postnatal day (P30D), and 60th postnatal day (P60D). The kidneys were harvested immediately following euthanasia for assays of histological examination, immunohistochemical staining (IHC), immunofluorescence staining, and western blotting. Body weight (BW) was recorded as mean ± SD g. Supplementary Figure 2: hyperoxia exposure attenuated membranous localization of ZO-1 in proximal tubules. (a) The distribution of N-cadherin and ZO-1 expression in glomeruli and proximal tubules of newborn rats, which were exposed to normoxia or hyperoxia from birth to 5th postnatal day (P5D) and 14th postnatal day (P14D), was measured, respectively, by immunofluorescence costaining (original magnification ×800. Scale bar, 40 μm. Arrow for membranous localization of ZO-1). (b) The distribution of ZO-1-associated nucleic acid-binding protein (ZONAB) and ZO-1 expression in glomeruli and proximal tubules of newborn rats, which were exposed to normoxia or hyperoxia from birth to 5th postnatal day (P5D) and 14th postnatal day (P14D), was measured, respectively, by immunofluorescent staining (original magnification ×800. Scale bar, 40 μm. Arrowheads for membranous localization of ZONAB). Supplementary Figure 3: neonatal hyperoxia downregulates expression of nephrin in glomeruli and that of Ki-67 in proximal tubules. Nephrin (a) and Ki-67 (b) expression in glomeruli and proximal tubules of newborn rats, which were exposed to normoxia or hyperoxia from birth to 5th postnatal day (P5D) and 14th postnatal day (P14D), was measured, respectively, by immunohistochemical staining (original magnification ×400. Scale bar, 20 μm). The box and whisker plot represents the immunostaining intensity of exp [file 2641461.f1.zip › 2641461/Supplementary materials/Supplemental figure 3.pptx]

## Slide 1
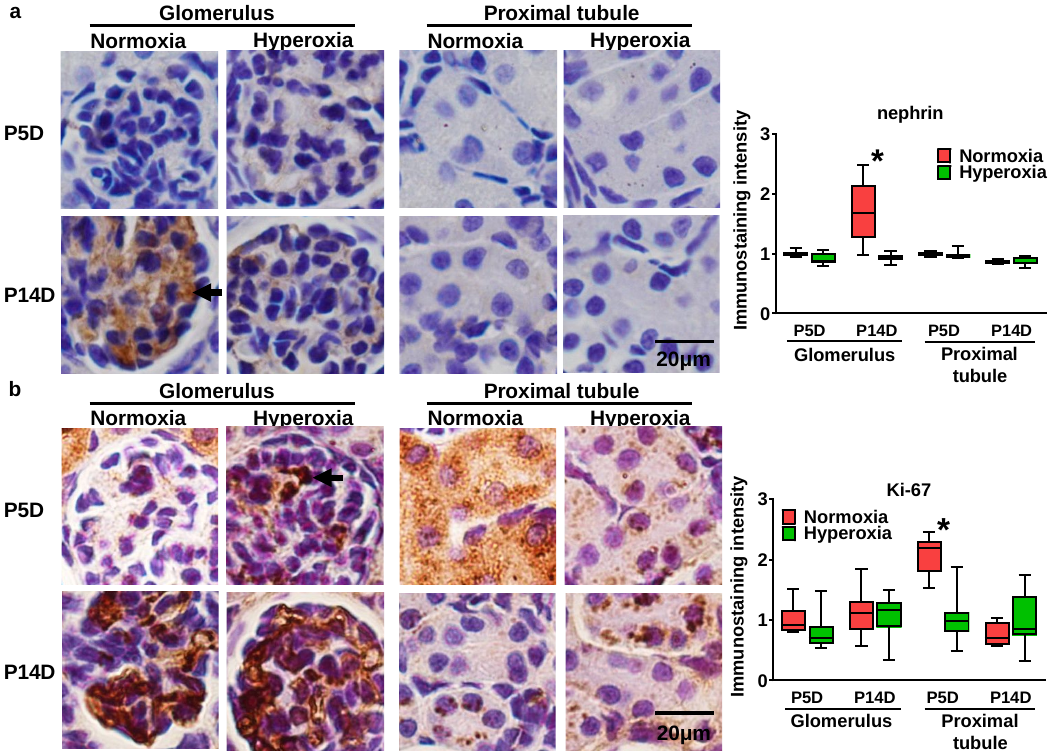

a
Glomerulus
Proximal tubule
Hyperoxia
Hyperoxia
Normoxia
Normoxia
P5D
P14D
20μm
b
Glomerulus
Proximal tubule
Hyperoxia
Hyperoxia
Normoxia
Normoxia
P5D
P14D
20μm
